# MiR210 in neonatal hypoxic-ischemic encephalopathy

**DOI:** 10.18632/oncotarget.14766

**Published:** 2017-01-20

**Authors:** Qingyi Ma, Lubo Zhang

**Affiliations:** Department of Basic Sciences, Center for Perinatal Biology, Division of Pharmacology, Loma Linda University School of Medicine, Loma Linda, CA, USA

**Keywords:** neonatal hypoxic-ischemic encephalopathy, mitochondrial dysfunction, microRNA 210, glucocorticoid receptor, iron-sulfur cluster scaffold protein

Neonatal hypoxic-ischemic encephalopathy (HIE) is associated with high neonatal mortality and severe long-term neurologic morbidity. The molecular mechanisms and the pathway of brain injury in infants with HIE remain largely elusive. Although therapeutic hypothermia is the current standard of care for newborns with moderate to severe HIE, nearly half of affected infants treated with hypothermia still die or suffer significant neurologic disability. Thus, there is an urgent need to further investigate the underlying mechanisms and to develop additional treatment strategies.

The clinical signs of neonatal HIE are associated with a primary energy failure followed by a series of neurotoxic events, including cytotoxic reaction, oxidative stress, proinflammatory response and the activation of multiple neuronal cell death pathways. Mitochondrial dysfunction occurs very early after neonatal HI and is regarded as the hallmark of neonatal HI brain injury [1]. The mitochondria electron transport chain is the primary contributor of excessive electrons resulting in the overproduction of reactive oxygen species (ROS) and oxidative stress in low oxygen conditions. For example, the mitochondrial respiratory complex I (NADH dehydrogenase) activity progressively declines during the first 24 hours of HI followed by the increase of oxidative stress. The permeability of mitochondrial outer membrane is increased, leading to the release of mitochondrial cytochrome *c* (cyt *c*) and subsequently the activation of caspase-dependent cell death. Considering the critical role of mitochondrial dysfunction in HI brain injury, further studies focusing on mitochondrial dysfunction and related cell death will advance our understanding of the molecular mechanisms underlying neonatal hypoxic-ischemic (HI) brain damage, and promote the development of potential therapeutic strategies in the treatment of infants with HI brain injury.

Several recent studies have demonstrated the critical role of microRNAs (miRs) in brain ischemic injury. MiRs are important players in the epigenetic control of gene expression, and silence gene expression by binding to the 3′-untranslated region (3′ UTR) of transcripts *via* their seed sequences at 5′ ends (nucleotides 2-8), resulting in transcript degradation or translational inhibition of the target genes. Among them, miR210 is *The Master Hypoxamir* of a specific group of miRs termed “*Hypoxamirs*” that are regulated by hypoxia. MiR210 has been implicated to play an important role in neural cell death. The recent study by Ma et al., 2016 [2] shed new light on the mechanisms of miR210 in neonatal HI brain injury. The study discovered in an animal model of neonatal rats that HI insults significantly increased miR210 levels in the brain. Of importance, inhibition of miR210 significantly reduced HI-induced neuronal death in the brain and improved long-term neurobehavioral function recovery, suggesting a detrimental role of miR210 in neonatal HI brain injury.

To get insight into the underlying mechanism of miR210 in neonatal HI brain injury, Ma et al. demonstrated a novel downstream target of the glucocorticoid receptor (GR) in the miR210-mediated effect [2]. In the neonatal rat brain, GR was highly expressed in the cortex and CA3 region of the hippocampus close to dentate gyrus, with somewhat lowered expression in the CA1 region. The intracerebroventricular injection of miR210 mimic significantly decreased GR protein abundance in the neonatal brain, and GR antagonist reversed the neuroprotective effect of miR210 inhibition in neonatal HI brain injury. This finding is consistent with other studies in which a local and direct activation of GR in the brain produced a neuroprotective effect in HI brain injury in neonatal rats [3,4]. The question remains as how GR protects the neonatal brain from HI insult. One of the possible mechanisms is that GR may prevent mitochondrial membrane permeability transition pore opening by increasing translocation of the anti-apoptotic protein Bcl-2 from the cytosol to the outer mitochondrial membrane in a GR-dependent manner [5], and inhibit the induction of caspase-dependent cell death pathway [6].

In addition to GR, iron-sulfur cluster scaffold protein (ISCU) is a key target of miR210, and may also engage in the mitochondrial dysfunction related neuronal death after neonatal HI brain injury. ISCU is a key chaperone protein for the assembly and transportation of cellular [4Fe-4S] and [2Fe-2S] iron-sulfur clusters to enzymes that are responsible for mitochondrial respiration and energy production, such as mitochondrial respiratory complexes I. Increasing evidence revealed that the miR-210-ISCU axis mediated the mitochondrial ROS production in response to hypoxic stress in different cell types, and was associated with cell death through the inhibition of complex I [7]. In agreement with this study, our ongoing work demonstrates that miR210 mimic injection into the brain downregulates ISCU protein in rat pups. Moreover, neonatal HI insult significantly reduces brain mitochondrial ISCU protein level and complex I activity, which are reversed by miR210 inhibition. Mounting evidence has shown that mitochondrial complex I deficiency is associated with a wide spectrum of neurodegenerative disorders through activation of mitochondria-dependent apoptotic molecular pathways. Two pathways have been proposed in initiating complex I deficiency-dependent neuronal apoptosis started from mitochondrial ROS generation [5,8]. Thus, complex I blockade stimulates intramitochondrial ROS, which is released into cytosol and damage the DNA strands, and increases the releasable soluble pool of cyt *c* within the mitochondrial intermembrane space, whereas activation of pro-cell death Bcl-2 family proteins is necessary to trigger neuronal death by increasing permeabilization of the outer mitochondrial membrane and releasing cytochrome *c* into the cytosol [5,8].

**Figure 1:**
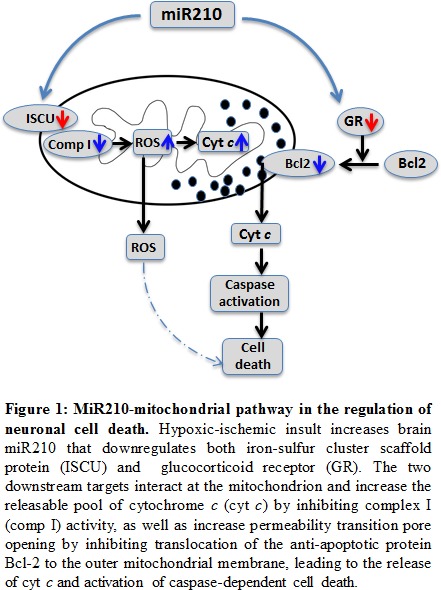
MiR210-mitochondrial pathway in the regulation of neuronal cell death Hypoxic-ischemic insult increases brain miR210 that downregulates both iron-sulfur cluster scaffold protein (ISCU) and glucocorticoid receptor (GR). The two downstream targets interact the mitochondrion and increase the releasable pool of cytochrome c (cyt c) by inhibiting complex I (comp I) activity, as well as increase permeability transition pore opening by inhibiting translocation of the anti-apoptotic protein Bcl-2 to the outer mitochondrial membrane, leading to the release of cyt c and activation of caspase- dependent cell death

In conclusion, studies by Ma et al., 2016 [2] reveal that miR210 is an upstream regulator of neuronal death after neonatal HI insult. A question of great interest is the extent to which the interaction of the two downstream targets of miR210, *i.e*. ISCU and GR, in the regulation of mitochondria-dependent apoptotic molecular pathways plays a key role in neonatal hypoxic-ischemic brain injury and longstanding neurobehavioral dysfunction (Figure 1). Further investigation is required to address this question and provides new insights in a novel target of epigenetic regulation in potential therapeutic strategies that may be beneficial for the treatment of infants with hypoxic-ischemic brain injury. This is of critical importance given the extreme limit of effective therapeutic intervention currently available other than hypothermia for this important clinical problem in newborns.
